# Analytical Profiling of Proanthocyanidins from *Acacia mearnsii* Bark and In Vitro Assessment of Antioxidant and Antidiabetic Potential

**DOI:** 10.3390/molecules23112891

**Published:** 2018-11-06

**Authors:** Xiao Chen, Jia Xiong, Shenlin Huang, Xun Li, Yu Zhang, Liping Zhang, Fei Wang

**Affiliations:** 1 Jiangsu Key Lab for the Chemistry and Utilization of Agro-Forest Biomass, College of Chemical Engineering, Nanjing Forestry University, Nanjing 210037, China; 13770765711@163.com (X.C.); shuang@njfu.edu.cn (S.H.); xunlee@163.com (X.L.); yuzhang@njfu.edu.cn (Y.Z.); 2 Food Bioprocessing and Nutrition Sciences Department, Plants for Human Health Institute, North Carolina State University, North Carolina Research Campus, Kannapolis, NC 28081, USA; jxiong5@ncsu.edu; 3 College of Materials Sciences and Technology, Beijing Forestry University, Beijing 100083, China; zhanglp418@163.com

**Keywords:** proanthocyanidins, HPLC/MS, MALDI-TOF/MS, antidiabetic, antioxidant, degree of polymerization

## Abstract

The proanthocyanidins from ethanol extracts (80%, *v*/*v*) of *Acacia mearnsii* (*A. mearnsii*) bark on chemical-based and cellular antioxidant activity assays as well as carbolytic enzyme inhibitory activities were studied. About 77% of oligomeric proanthocyanidins in ethanol extracts of *A. mearnsii* bark were found by using normal-phase HPLC. In addition, HPLC-ESI-TOF/MS and MALDI-TOF/TOF MS analyses indicated that proanthocyanidins from *A. mearnsii* bark exhibited with a degree of polymerization ranging from 1 to 11. These results of combined antioxidant activity assays, as well as carbolytic enzyme inhibitory activities of proanthocyanidins from *A. mearnsii* bark, indicated an encouraging antioxidant capacity for the high polyphenol content and a potential for use as alternative drugs for lowering the glycemic response.

## 1. Introduction

Proanthocyanidins (PAs), a family of plant polyphenols, are secondary metabolites of plants, which are important for plant growth and protection against infection and injury [1]. Different types of PAs exist based on the substitution pattern of monomeric flavan-3-ol units linked by a single C_4_–C_8_ or C_4_–C_6_ bond (B-type PAs) or by an additional C_2_–O–C_7_ or C_2_–O–C_5_ bond (A-type PAs) [2]. They are widely distributed in plant resources, usually comprising procyanidins, prodelphinidins, and propelargonidins, which consist of (epi)catechin, (epi)gallocatechin, and (epi)afzelechin units, respectively [3,4].

Many plant resources have been found to be rich in PAs, which are present in their barks, branches, leaves, seed coats, and fruits [5,6]. Consequently, many researchers have studied their health benefits [7,8]. In addition, they have been reported to exert notable pharmacological effects, including antioxidative, antimicrobial, antiproliferative, enzyme inhibitory, and cardioprotective effects [5,9]. The length of PA chain, or the degree of polymerization (DP), appears to play a pivotal role in bioactivity [10], and varies between different types of PAs. Smaller PAs are more effective as superoxide anion scavengers, free-radical scavengers, and xanthine oxidase inhibitors than larger PAs, whereas larger PAs slow intestinal α-glucose absorption by inhibiting intestinal α-glycosidase activity more effectively. Oligomeric PAs may inhibit proliferation more effectively than monomeric PAs [11].

The PAs from *Acacia mearnsii* (*A. mearnsii*) bark were used to produce vegetable-tanned leather [12]. Many researchers have studied its chemical composition, DP, and biological activities. Previously published research indicated that most of flavan-3-ol monomers consist of catechin, gallocatechin, fisetinidol, and robinetinidol, and the maximum molecular weight (MW) of PAs observed by matrix-assisted laser desorption/ionization-time-of-flight mass spectrometry (MALDI-TOF-MS) was 2333 Da (octamers) [13,14]. The MW of PAs in *A. mearnsii* bark reported may range from 300 Da for monomeric flavan-3-ol up to 3000 Da for polymeric PAs [15]. Additionally, the composition, characterization, and bioactivity evaluation of the *A. mearnsii* bark PAs have been reported in our group [7,16,17].

The purpose of this work was to characterize the DP and MW distribution of PAs in the *A. mearnsii* bark, and then study its antioxidant activities by using a combined method of chemical-based and cellular antioxidant activity. Simultaneously, we uncovered the potential for its use as antidiabetic ingredients by detecting its inhibitory effects on carbolytic enzyme. 

## 2. Results and Discussion

### 2.1. Determination of Total Polyphenol Content (TPC), PAs, and Antioxidant Activity

As measured by the Folin-Ciocalteu method and vanillin assay, the concentrations of polyphenols and PAs in *A. mearnsii* bark extract (ABE) was 589 mg GAE/g dried extract and 215 mg catechin/g dried extract. The chemical-based antioxidant capacity of ABE was measured by using the assays of DPPH, ABTS, ORAC, and PSC. The assays of DPPH, ABTS and PSC to measure the ABE possessed a good effect on scavenging free radical with the IC_50_ of 61.5 ± 0.5 μg/mL, 23.3 ± 0.3 μg/mL and 7.6 ± 0.3 μg/mL, respectively. The Trolox equivalent antioxidant capacity (TEAC) values were used in DPPH, ABTS, and ORAC assays, calculated as mg Trolox equivalent (TE)/g dried extract. DPPH, ABTS, and ORAC confirmed the encouraging antioxidant capacity of ABE with 1220 ± 8, 1820 ± 4, 2739 ± 14 mg TE/g dried extract, explained by its richness in polyphenol compounds, because that polyphenols were found to contribute effectively to the antioxidant activity of many plants [18].

### 2.2. Normal-Phase HPLC Analysis

**Figure 1:**
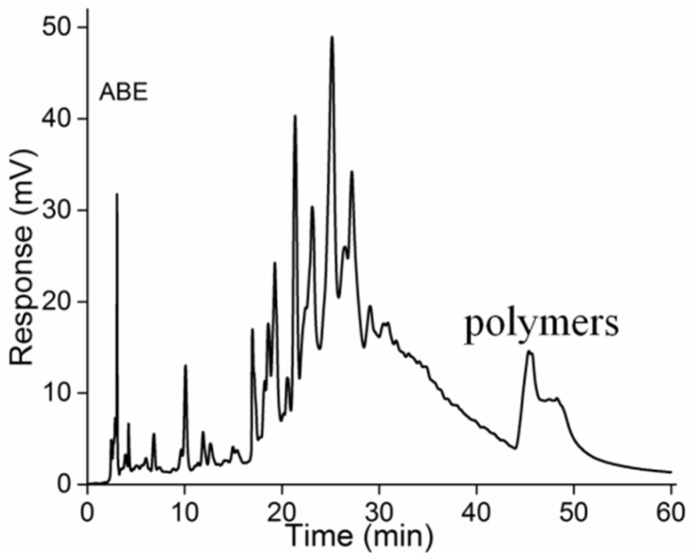
Representative normal-phase HPLC/Variable Wavelength Detector (VWD) chromatograms of PAs from ABE.

Previous studies have shown that larger PAs can be separated based on their DP by a silica column [17,19]. A normal-phase HPLC method was developed to analyze PAs from *A. mearnsii* bark. Because there was no normal-phase HPLC/MS, we were unable to identify each peak in the chromatogram presented in Figure 1. HPLC chromatography showed that ABE possessed the very high content of oligomers due to its strong signal intensity between 20 min and 30 min, but a small hump at about 45 min which may represent the polymeric PAs. Meanwhile, other researchers also have reported that ABE consisted of approximately 9% monomers, 42% dimers, 40% trimers, 9% tetramers, and 1% pentamers, with a higher proportion of oligomers by mass [12]. However, ESI work poorly for detecting PAs with a DP greater than 4; consequently, we considered that NP-HPLC chromatogram may indicate an accurate results with about 77% oligomers in ABE. Additionally, it was feasible to characterize all the MWs of PAs from *A. mearnsii* bark. 

### 2.3. RP-HPLC/MS Analysis

**Figure 2:**
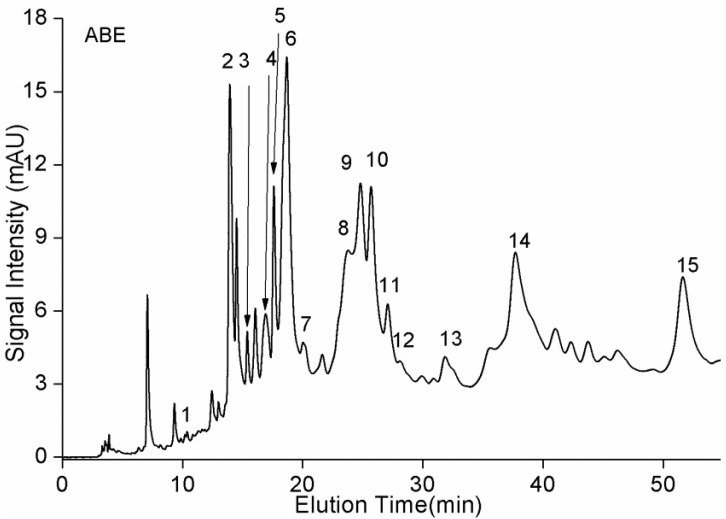
Representative reverse-phase HPLC/VWD chromatograms of PAs from ABE.

A method of reverse-phase HPLC was conducted to identify oligomeric PA monomers, dimers, and trimers (Figure 2), and their results of mass spectra were presented in Supplementary Materials (Figure S1). Based on our previous work [17], their proposed compounds were displayed in Table 1. As shown in Table 1, the results indicated that no A-type PAs were tentatively identified, and PAs with a DP greater than 3 were also not detected. Moreover, epicatechin was not detected in *A. mearnsii* bark, and it was also not found in ABE by previously published reports [12,17,20]. In the ESI spectrum, groups of peaks were separated by intervals of *m*/*z* 272, 288, and 304, corresponding to the incremental mass of fisetinidol, catechin or robinetinidol, and gallocatechin extension, respectively. Many of the identified components were isomers, six dimers and seven trimers with different MWs found in ABE, which might be constituents of fisetinidol, catechin or robinetinidol, and gallocatechin linked by C_4_–C_6_ or C_4_–C_8_ [17]. Among the 15 components, gallate units were not tentatively identified. Catechin and gallocatechin were identified by comparing with standards.

### 2.4. MALDI-TOF/TOF-MS Analysis of ABE

About 77% of PAs were considered as oligomers; meanwhile, MALDI is superior to ESI for investigating polymeric PAs. MALDI-TOF/TOF-MS all worked well in other work [21,22], and Cs seems to work better for detecting PAs with a DP greater than eight [22]. Therefore, we used MALDI-TOF/TOF-MS with cationizing agent Cs^+^ to analyze PAs in ABE. Figure S2 (Supplementary Materials) showed the MALDI-TOF mass spectra of the PAs from ABE, recorded as Cs^+^ adducts in the positive ion reflectron mode. The identity of these compounds was established by comparison of the observed [M + Cs]^+^ with the theoretical values calculated according to the formula with some modification [22]:

[M + Cs]^+^ = 290.08 × C/R + 274.08 × F + 306.07 × GC + 152.01 × GAL − 2.02 × B − 4.04 × A + 132.91 (1)

where C, R, F, GC, and GAL correspond to the numbers of catechin, robinetinidol, fisetinidol, gallocatechin, and galloyl moieties, respectively; and A and B correspond to the numbers of A and B linkages.

Many PAs were detected in ABE (Table 2), corresponding to a wide variety of structures from trimers to undecamers of B-type PAs, and none of them were galloylated. All of the PAs detected by MALDI-TOF/TOF-MS might be constituents of catechin, fisetinidol, robinetinidol, and gallocatechin linked by C_4_–C_6_ or C_4_–C_8_. However, the order of the linkage was not deduced and PAs with a DP greater than 11 were not detected. Based on the analysis of ESI/MS and MALDI-TOF/MS, polyphenol in ABE consists of PAs, of which molecular masses range from 290 Da to 3300 Da. PAs in ABE were constituents of procyanidins, profisetinidin, prorobinetinidin, and prodelphinidins without galloylated. It was not common because profisetinidin and prorobinetinidin are less common in nature, due to the fact that the most widely distributed PAs in plants contain either exclusively procyanidins or a mixture of procyanidins and prodelphinidins (heterogeneous PAs), with prodelphinidins also being less common in nature than procyanidins. Meanwhile, the absence of 5-hydroxy groups of profisetinidin and prorobinetinidin in the chain extender unit of ABE imparts stability to the interflavanyl bond against acid hydrolysis, which increased the difficulty of the determination of PAs in ABE [12,23]. On the other hand, the difference was that the PAs from many foods seem to possess a bigger MW, such as grape seed PAs with a DP than 24, grape skin PAs with a DP than 39, and *Hop* PAs with a DP than 24, detected by acid catalysis in the presence of phloroglucinol and gel permeation chromatography [19]. Undoubtedly, the MW determines PAs bioavailability and bioactivity; therefore, we think it should be further studied for the biological activities.

### 2.5. α-Amylase and α-Glucosidase Inhibition

**Figure 3:**
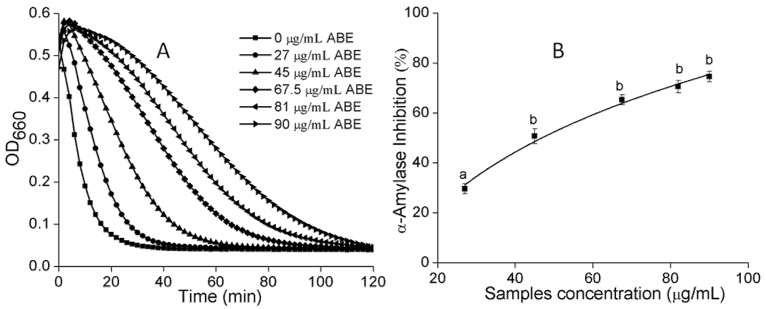
(**A**) The kinetic curves of starch hydrolysis by α-amylase in the presence of different concentrations of ABE; (**B**) Dose–response curve of the α-amylase inhibitory activity of PAs from ABE. Values labeled with the same letter (a–b) are not significantly different from each other (*p* < 0.05). The symbols represent mean ± SD.

**Figure 4:**
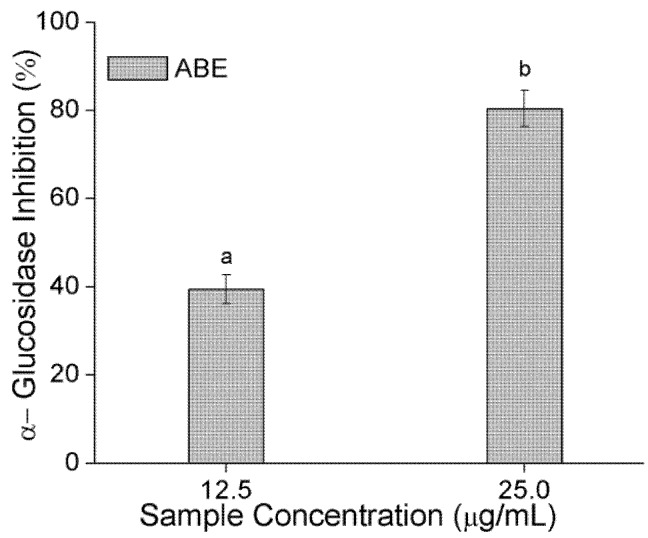
Effect of PAs from ABE on the inhibition of α-glucosidase activity. Values labeled with different letters (a–b) are significantly different from each other (*p* < 0.05). The symbols represent mean ± SD.

α-Amylase and α-glucosidase are key enzymes involved in the breakdown of starch and intestinal absorption, respectively. Consequently, inhibition of α-amylase and α-glucosidase have been suggested as a preventive antidiabetic therapy [24,25]. In an earlier stage, the water and ethyl acetate fractions were extracted from ABE. The water fraction was found to discriminate better than the ethyl acetate fraction against α-amylase and α-glucosidase (4.4- and 2.1-folds higher, respectively). However, the contents of PAs and polyphenols in the ethyl acetate fraction were richer than those in the water fraction (about 1.2- and 1.5-folds higher, respectively). Consequently, our preliminary studies revealed that water is suitable for extracting PAs as an alternative drug for antidiabetes drug. We also found the same results that PAs with a greater DP showed clearer inhibition against α-amylase at a lower concentration [24]. As shown in Figure 3A, based on the dose response curves, the IC_50_ (Figure 3B) of ABE against α-amylase was 45.83 μg/mL. Previous work also found the active substances are PAs oligomers mainly composed of 5-deoxyflavan-3-ol units, such as robinetinidol and fisetinidol [14]. Meanwhile, PAs were a mixed noncompetitive inhibitor against α-amylase activity [24]. ABE could inhibit 40% α-glucosidase at a low concentration, shown in Figure 4. The PAs from ABE showed a stronger inhibitory effect on α-glucosidase than α-amylase, which might because PAs inhibited α-amylase activity mainly contributed by DP [24]. Notably, this ability of any PAs to inhibit digestive enzymes is of particular importance, as bioavailability is not needed for PAs of any size to inhibit the digestion of lipids or carbohydrates in the gastrointestinal lumen [11]. As a reference, the IC_50_ values of acarbose, measured under the same conditions, were 8.25 μg/mL for α-amylase and 164.21 μg/mL for α-glucosidase. These values were very close to those of published work [25,26,27]. Compared to acarbose, the ABE exhibited a strong inhibitory effect on carbolytic enzyme. ABE may have the potential as an effective therapy for postprandial hyperglycemia with minimal side effects, because only mild α-amylase inhibition is recommended to prevent the abnormal bacterial fermentation of undigested carbohydrates in the colon [28,29].

### 2.6. Cellular Antioxidant Activity (CAA)

The existing chemical methods to determine antioxidant activity do not indicate the accurate results, because metabolism and bioavailability are out of consideration. Moreover, the cells, tissue, and organs are unlikely to be in vivo exposed to the PAs-rich ethanol extract in the concentrations and chemical structures tested, since metabolic transformation after digestion and absorption will change the parent structures of PAs from ABE [17]. The CAA assay reflected the cellular physiological conditions and considered the bioavailability and metabolism issues, and it seems to provide a more accurate measure of bioactivities in biological systems [30]. Moreover, it is becoming unacceptable for manuscripts based solely on colorimetric methods in some journals, and it is encouraged to determine bioactive compound capability in in vitro biological test(s), such as cell lines [31]. Our previously published files focused on chemical-based antioxidant activity [16,17]; consequently, we thought a work of combination of chemical-based methods and cellular-based assay really needed to be conducted. 

**Figure 5:**
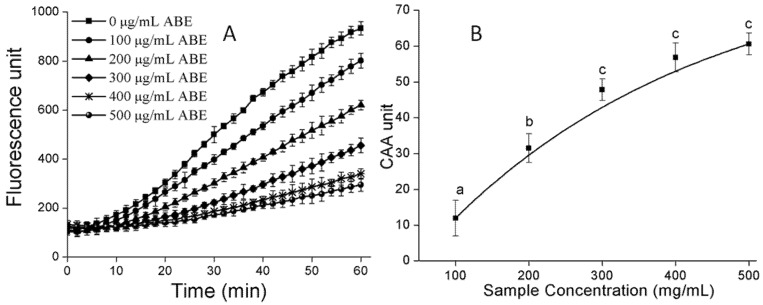
(**A**) Peroxyl radical-induced oxidation of DCFH to DCF in HepG2 cells and the inhibition of oxidation by PAs in ABE over time; (**B**) Dose–response curves for inhibition of peroxyl radical-induced DCFH oxidation by ABE. Values labeled with the same letter (a–c) are not significantly different from each other (*p* < 0.05). The symbols represent mean ± SD.

The dose–response curves, generated from data obtained in Figure 5A for ABE, were shown in Figure 5B. In addition, the CAA EC_50_ value was 0.3 ± 0.003 mg/mL. The results indicated that the EC_50_ of the ABE determined by the cellular-based method was much higher than those determined by the chemical-based methods of DPPH, ABTS and PSC with the IC_50_ values of 61.5 ± 0.5 μg/mL, 23.3 ± 0.3 μg/mL and 7.6 μg/mL, respectively. This contradiction may exhibit the low bioaccessibility rate of PAs, which was consistent with the approximately 17.9% accessibility rate of ABE detected by the method of in vitro digestion [17]. The PAs with large molecular dimensions (DP > 4) cannot easily enter modeled endothelial cells, mainly due to the fact that PAs were unlikely to pass through the lipid bilayer via the transcellular pathway due to the large number of hydrophilic hydroxyl groups [32,33]; therefore, a larger size of PAs may show a lower bioaccessibility rate. We considered that a plant rich in PAs with lower DP deserved attention.

## 3. Materials and Methods

### 3.1. Materials and Reagents

*A. mearnsii* bark was kindly supplied by Crown Forest Farm in Guangxi Province (Guangxi, China) identified by Prof. F. Wang, Nanjing Forestry University. (+)-Catechin (purity > 97% by HPLC), gallocatechin (purity > 97% by HPLC), acarbose (purity > 99% by HPLC) 2,2′-azinobis-(3-ethylbenzthiazoline-6-sulphonate) (ABTS), ascorbic acid (purity > 99% by HPLC), 2,2-diphenyl-1-picrylhydrazyl (DPPH), α-amylase (type VI-B, from porcine pancreas), α-glucosidase (from *Saccharomyces cerevisiae*), p-nitrophenyl-α-D-glucopyranoside (pNPG), 2,5-dihydroxy benzoic acid (DHB), and cesium triflouroacetate were purchased from Sigma-Aldrich (St. Louis, MO, USA). Gallic acid (purity > 97% by HPLC) and 2′,7′-dichlorofluorescin diacetate (DCFH-DA) were purchased from Macklin (Shanghai, China). Trolox (purity > 99% by HPLC) and trifluoroacetic acid (TFA) were obtained from Aladdin (Shanghai, China). Fluorescein sodium and 2,2′-Azobis(2-amidinopropane) dihydrochloride (AAPH) were purchased from Energy Chemical Company Ltd. (Shanghai, China). Minimum essential medium (MEM) and HepG2 cells were acquired from KeyGen Biotech Company Ltd. (Nanjing, China). Fetal bovine serum (FBS) was purchased from Zhejiang Tianhang Biological Technology Company Ltd. (Zhejiang, China). Dichloromethane, acetic acid, and methanol (all chromatographic pure) were purchased from Tedia (Fairfield, OH, USA). Other reagents were of analytical grade.

### 3.2. Sample Extraction

The dried raw material was ground using a grinder. The powder was then passed through a #5 mesh, with a sieve opening of 4 mm. The obtained powder (25 g) was defatted twice with hexane (solid/solvent, 1:5, *w*/*v*) and stirred at 300 rpm. Defatted samples were suspended in 80% (*v*/*v*) ethanol (250 mL) and stirred at 300 rpm under room temperature. The extraction was performed three times, and the corresponding extraction times were 4, 3, and 2 h, respectively. Then, the combined extract was evaporated to remove the organic solvent. Finally, the aqueous phase was lyophilized to obtain the *A. mearnsii* bark extract. The obtained extracts were stored at 4 °C until further analysis.

### 3.3. Determination of PAs and TPC

The PAs content was determined according to a modified vanillin assay. Briefly, 2.5 mL of 3% (*w*/*v*) vanillin in methanol and 2.5 mL of 30% (*v*/*v*) H_2_SO_4_ in methanol were added to 0.5 mL of the prepared solution with ABE to perform the vanillin reaction at room temperature for 20 min under dark conditions. Methanol was used as a blank. The absorbance was measured at 500 nm using a Shimadzu UV-2450 and compared to that of the prepared blank. The contents of PAs were expressed as mg catechin equivalents/g dried extract.

The TPC was determined using the Folin–Ciocalteu assay. In brief, 0.5 mL of the prepared solution with ABE was added to 2.5 mL of 10% Folin–Ciocalteu reagent. Then, 2 mL of 7.5% Na_2_CO_3_ (*w*/*v*) was added to the mixture, which was allowed to stand for 120 min at ambient temperature in the dark. Finally, the absorbance of the mixture was measured at 760 nm using a Shimadzu UV-2450. The results were expressed as mg gallic acid equivalents (GAE)/g dried extract.

### 3.4. NP-HPLC/ Variable Wavelength Detector (VWD) Analysis

Based on our previous method [17], a normal-phase HPLC Agilent 1260 HPLC-VWD platform was used to analyze the DP distribution of PAs in ABE.

### 3.5. RP-HPLC/MS Analysis

The extracts were dissolved in methanol at 5 mg/mL and then filtered through a 0.22 μm membrane and analyzed on a reverse-phase Agilent 1260 HPLC-DAD platform with an injection volume of 5 μL. Separation was performed on a Zorbax Eclipse XDB-C18 (Agilent, Santa Clara, CA, USA) column (250 × 4.6 mm, 5 μm) at 30 °C. According to a previous reference [34], mobile phase A was 0.25% acetic acid in water, and mobile phase B was 0.25% acetic acid in water and acetonitrile (50:50, *v*/*v*) at a flow rate of 0.7 mL/min, using a modified gradient program as follows: 10–27% B (0–9 min), 27–27.7% B (9–14 min), 27.7–28% B (14–30 min), 28–40% B (30–60 min), and 40–100% B (60–65 min). The conditions for MS spectrometric detection in ESI mode were as follows: negative ion mass spectra of the column eluate were recorded in the range of m/z 50–2000 at a scan speed of 13,000 m/z/s. Nitrogen was used as a drying gas at a flow rate of 12.0 L/min and as a nebulizing gas at pressures of 35.0 psi. The nebulizer temperature was set at 365 °C.

### 3.6. MALDI-TOF/TOF-MS Analysis of ABE

The MALDI-TOF mass spectra were performed on an MALDI-TOF instrument (Ultraflextreme, Bruker, Karlsruhe, Germany). The experimental conditions used were: pulsed-ion extraction (PIE) delay: 130 ns, ion source voltage 1: 20 kV, ion source voltage 2: 17.8 kV, lens voltage: 8 kV, linear detector voltage: 2.703 kV, laser repetition rate: 1000 Hz, and number of shots: 1500. Mass spectra were collected by averaging the signals of at least 500 laser shots over the m/z range of 500–5000. Based on a previous study [22], the samples were dissolved in methanol (5 mg/mL). The sample solution was mixed with a 1% aqueous TFA solution consisting of DHB (10 mg/mL) and cesium triflouroacetate (1 mg/mL).

### 3.7. Chemical Antioxidant Activity

#### 3.7.1. DPPH and ABTS Assays

DPPH and ABTS assays were performed following our previously published methods [17].

#### 3.7.2. Oxygen Radical Absorbance Capacity (ORAC) Assay

The ORAC assay was conducted as the previously described method with some improvements [27]. The fluorescein sodium salt and AAPH were prepared fresh and dissolved in 75 mM NaH_2_PO_4_–Na_2_HPO_4_ buffer (pH 7.4) to give concentrations of 0.96 and 119 mM, respectively. The assay was performed in a 96-well microplate as follows: 20 μL of samples or Trolox solution were added to a well, and then 200 μL of fluorescein sodium salt was added. After incubation for 20 min at 37 °C, 20 μL of evocating agent (AAPH) was added; 75 mM phosphate (pH 7.4) was used as a blank. After thoroughly shaking for 5 s, the fluorescence intensity was recorded every 5 min for 35 cycles at 37 °C, with 538 nm as the emission wavelength and 485 nm as the excitation wavelength. All tests were performed in triplicate and averaged. The results were expressed in mg Trolox/g dried extract.

#### 3.7.3. Assay of Rapid Peroxyl Radical Scavenging Capacity (PSC)

The antioxidant activity of PAs from ABE was measured using the PSC assay [35]. The assay was carried out as follows: 100 μL of samples or positive control were added to a black 96-well plate, and then 100 μL DCFH dye (33.06 μM) was added, for which 1 mM KOH was used to remove the diacetate moiety of DCFH-DA for 5 min before use. Then, 50 μL of AAPH (40 mM) was added, and the reaction was performed at 37 °C for 34 min. Fluorescence values were recorded at the 485-nm excitation wavelength and 538-nm emission wavelength. Phosphate (75 mM, pH 7.4) was used as a blank.

### 3.8. α-Amylase and α-Glucosidase Inhibition

The inhibitory activities of ABE against α-amylase were determined using the turbidity measurement as previously described [24]. Sodium phosphate buffer (0.1 M, pH 6.9) was used as a solvent. Corn starch was dissolved in buffer at 20 mg/mL, followed by gelatinizing at 100 °C for 2.5 min. The samples or positive were diluted in buffer to provide five gradient concentrations. An α-Amylase solution (4 U/mL in buffer 20 μL) was pre-incubated with an inhibitor solution (20 μL) with a series of concentrations in a 96-well microplate and kept at 37 °C for 15 min. The reaction was initiated by injecting 60 μL of the gelatinized corn starch solution. The turbidity changes were recorded at 660 nm every 2 min for 60 cycles at 37 °C. The percentage of inhibition was calculated as follows:

Inhibition (%) = [(*AUC*_sample_ − *AUC_control_*)/*AUC_sample_*] × 100 (2)

where *AUC_sample_* is the area under the inhibitory curve; and *AUC_control_* is the area under the control curve. IC_50_ can be defined as the concentration of an inhibitor that induces 50% inhibition of enzymatic activity under specified assay conditions. This value was obtained from interpolation of the percentage of inhibition against an inhibitor concentration curve.

The inhibitory effect on α-glucosidase was performed with our previously described method [17].

### 3.9. Cell Culture and Treatment

HepG2 were incubated in an MEM solution containing 10% FBS and 1% *v*/*v* penicillin–streptomycin. The cells were maintained at 37 °C in an incubator with 5% CO_2_. All cells used in this study were between passages 15 and 35.

### 3.10. Cytotoxicity

The cytotoxicity of the PAs from ABE on HepG2 cells was determined as previously described, with slight modifications [36]. First, 4 × 10^4^ HepG2 cells/well were grown in a 96-well plate containing 100 μL growth medium (MEM containing 10% FBS, 1% (v/v) penicillin–streptomycin). After culturing for 24 h in an incubator, the growth medium was removed and cells were washed with PBS. The ABE was dissolved in DMSO and diluted to a maximum concentration (500 μg/mL) with MEM (excluding FBS), and then 100 μL was added to a 96-well plate (0.5% DMSO). The cells were incubated at 37 °C with 5% CO_2_ for a further 24 h. Following the incubation, the growth medium was removed, and the cells were washed with PBS. Then, 100 μL of growth medium and 10 μL of detection reagent MTT (4 mg/mL) were added to the cells. After incubating for 4 h, the previous solution medium was removed and 150 μL DMSO was added. The plates were shaken for 10 min to dissolve purple crystals and measured at 540 nm using a 96-well plate reader. The sample concentrations that decreased absorbance readings by <10% compared with the control were considered non-cytotoxic and used to determine CAA.

### 3.11. Cellular Antioxidant Activity

The CAA assay was performed as previously described, with some modifications [37]. Briefly, HepG2 cells were seeded at a density of 6 × 10^4^ cells per well in a black 96-well microplate in 100 μL of growth medium/well. Twenty-four hours after seeding, the growth medium was removed, and the cells were washed with 100 μL of PBS. Wells were then exposed to 100 μL of treatment medium containing control extract, or test extract (100–500 μg/mL) plus 25 μM DCFH-DA for 1 h. A 20 mM DCFH-DA stock solution was prepared in methanol and stored at −20 °C until use. Then, each well was washed using 200 μL PBS, and 600 μM of AAPH was applied to the cells in 100 μL HBSS, which was dissolved for 1 h before used. The absorbance of the 96-well microplate was detected every 2 min for 1 h, using an ELISA reader with emission at 538 nm and excitation at 485 nm. Each plate included a triplicate control and blank wells; control wells contained cells treated with DCFH-DA and oxidant; blank wells contained cells treated with dye and PBS without oxidant. 

### 3.12. Statistical Analysis

The sample was analyzed in triplicate. Data were expressed as mean ± SD. Significant differences between the means of parameters were determined by using SPSS 20 statistical software (SPSS, Chicago, IL, USA) (*p* < 0.05). All the figures were fabricated by using OriginPro8 software (OriginLab, Northampton, MA, USA).

## 4. Conclusions

In the present study, about 77% of oligomers and PAs with MWs ranging from 290 Da to 3300 Da in ethanol extracts of *A. mearnsii* bark were firstly identified. Moreover, in vitro analyses for the antioxidant and carbolytic enzyme inhibitory effects of extracts from *A. mearnsii* bark indicated that it has biological activities related to diabetes. In other research involving diabetic mice, B-type oligomeric PAs act more effectively than those of A-type oligomers in lowering fasting blood glucose, oligomeric PAs with a mean DP reduce blood glucose about three times more effectively, compared to polymeric PAs [11]; meanwhile, antidiabetic effects of PAs from *A. mearnsii* bark are attributable not only to the absorbable constituents but also to the non-absorbable constituents that play roles in the gastrointestinal tract [38]. These results exhibited that the PAs from *A. mearnsii* bark may have a potential for use in functional drug applications aimed at lowering the glycemic response with few side effects and eliminating the free radical in vivo. However, further research is still needed to isolate these bioactive compounds and conform to the biological effect through animal and clinical trials.

## Figures and Tables

**Table 1:** Components identified in the ABE by LC/MS.

Approximate Retention Time from VWD (min)	Peak	Mass (Da)	Proposed Structure
10.4	1	306	gallocatechin
14.0	2	594	B dimer
15.6	3	290	catechin
17.1	4	578	B dimer
17.8	5	578	B dimer
18.7	6	578	B dimer
19.9	7	866	B trimer
23.3	8	882	B trimer
24.4	9	562	B dimer
25.3	10	882	B trimer
26.9	11	866	B trimer
28.0	12	562	B dimer
33.6	13	866	B trimer
39.2	14	866	B trimer
52	15	850	B trimer

Catechin and gallocatechin supported by analysis of standard solutions.

**Table 2:** PAs detected by matrix-assisted laser desorption/ionization-time-of-flight mass spectrometry from ABE.

Polymer	Number of C/R	Number of GC	Number of F	Number of GAL	Calculated [M + Cs]^+^	Observed [M + Cs]^+^
Trimer	1	0	2	0	967.1	967.43
	2	0	1	0	983.1	983.43
	3	0	0	0	999.1	999.43
	2	1	0	0	1015.1	1015.43
Tetramer	1	0	3	0	1239.2	1239.57
	2	0	2	0	1255.2	1255.58
	3	0	1	0	1271.2	1271.58
	4	0	0	0	1287.2	1287.58
	3	1	0	0	1303.2	1303.58
	2	2	0	0	1319.2	1319.58
Pentamer	2	0	3	0	1527.2	1527.72
	3	0	2	0	1543.2	1543.72
	4	0	1	0	1559.2	1559.72
	5	0	0	0	1575.2	1575.73
	4	1	0	0	1591.2	1591.73
	3	2	6	0	1607.2	1607.73
Hexamer	3	0	3	0	1815.3	1815.86
	4	0	2	0	1831.3	1831.86
	5	0	1	0	1847.3	1847.87
	6	0	0	0	1863.3	1863.87
	5	1	0	0	1879.3	1879.86
	4	2	0	0	1895.3	1895.86
Heptamer	4	0	3	0	2103.3	2103.99
	5	0	2	0	2119.3	2119.99
	6	0	1	0	2135.3	2135.99
	7	0	0	0	2151.3	2151.99
	6	1	0	0	2167.3	2167.99
	5	2	0	0	2183.3	2183.99
Octamer	5	0	3	0	2391.4	2392.12
	6	0	2	0	2407.4	2408.12
	7	0	1	0	2423.4	2424.12
	8	0	0	0	2339.4	2440.12
	7	1	0	0	2455.4	2456.12
	6	2	0	0	2471.4	2472.12
Nonamer	7	0	2	0	2695.5	2697.27
	8	0	1	0	2711.5	2714.29
	9	0	0	0	2727.5	2729.30
	8	1	0	0	2743.5	2745.28
Decamer	7	0	3	0	2969.5	2969.38
	8	0	2	0	2985.5	2985.40
	9	0	1	0	3003.5	3002.42
	10	0	0	0	3015.5	3016.42
	9	1	0	0	3031.5	3033.41
Undecamer	10	0	1	0	3287.6	3288.59
	11	0	0	0	3303.6	3304.59

C, R, F, GC, and GAL represent catechin, robinetinidol, fisetinidol, gallocatechin, and galloyl moieties, respectively.

## Supplementary Materials

The following are available online, Figure S1: Mass spectrometry and proposed compounds of PAs from *A. mearnsii* bark under different retention times. Figure S2: MALDI-TOF positive reflectron mode mass spectra of ABE: full spectrum (A), details of the 500–1000 m/z (B), 1100–1900 m/z (C), and 2000–3600 m/z (D).
