# Supplementary material for: Analytical Profiling of Proanthocyanidins from Acacia mearnsii Bark and In Vitro Assessment of Antioxidant and Antidiabetic Potential
Source: Molecules. 2018 Nov 6;23(11):2891. doi: 10.3390/molecules23112891 (PMC6278516; doi:10.3390/molecules23112891)
Supplement: Supplementary file 1 [file molecules-23-02891-s001.pdf]

molecules

Analytical Profiling of Proanthocyanidins from *Acacia mearnsii* Bark and In Vitro Assessment of Antioxidant and Antidiabetic Potential

Xiao Chen [1], Jia Xiong [2], Shenlin Huang [1], Xun Li [1], Yu Zhang [1], Liping Zhang [3] and Fei Wang [1,*]

[1] Jiangsu Key Lab for the Chemistry and Utilization of Agro-Forest Biomass, College of Chemical Engineering, Nanjing Forestry University, Nanjing 210037, China; 13770765711@163.com (X.C.); shuang@njfu.edu.cn (S.H.); xunlee@163.com (X.L.); yuzhang@njfu.edu.cn (Y.Z.)
[2] Food Bioprocessing and Nutrition Sciences Department, Plants for Human Health Institute, North Carolina State University, North Carolina Research Campus, Kannapolis, NC 28081, USA; jxiong5@ncsu.edu
[3] College of Materials Sciences and Technology, Beijing Forestry University, Beijing 100083, China; zhanglp418@163.com
* Correspondence: hgwf@njfu.edu.cn; Tel.: +86-25-85427649

Academic editors: Margarida Castell Escuer and Mariona Camps-Bossacoma
Received: 16 October 2018; Accepted: 1 November 2018; Published: date

Abstract: The proanthocyanidins from ethanol extracts (80%, *v*/*v*) of *Acacia mearnsii* (*A. mearnsii*) bark on chemical-based and cellular antioxidant activity assays as well as carbolytic enzyme inhibitory activities were studied. About 77% of oligomeric proanthocyanidins in ethanol extracts of *A. mearnsii* bark were found by using normal-phase HPLC. In addition, HPLC-ESI-TOF/MS and MALDI-TOF/TOF MS analyses indicated that proanthocyanidins from *A. mearnsii* bark exhibited with a degree of polymerization ranging from 1 to 11. These results of combined antioxidant activity assays, as well as carbolytic enzyme inhibitory activities of proanthocyanidins from *A. mearnsii* bark, indicated an encouraging antioxidant capacity for the high polyphenol content and a potential for use as alternative drugs for lowering the glycemic response.

Keywords: proanthocyanidins; HPLC/MS; MALDI-TOF/MS; antidiabetic; antioxidant; degree of polymerization

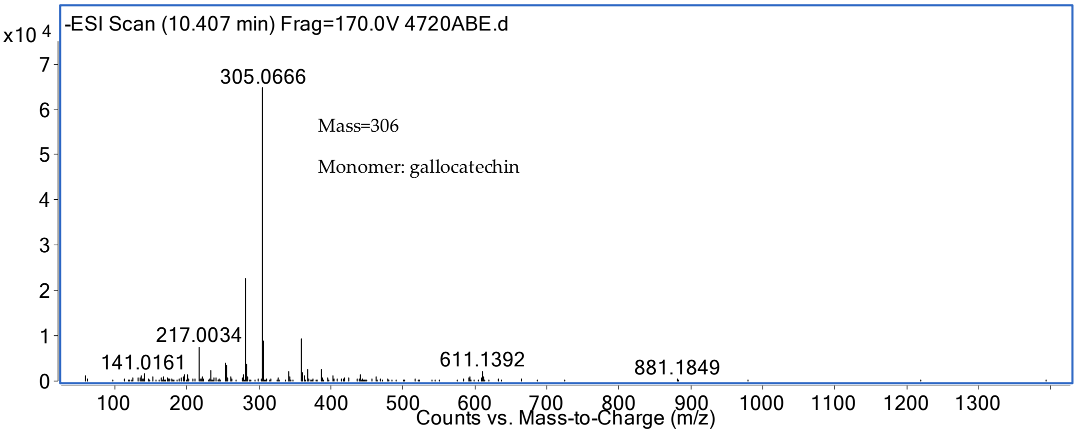
-ESI Scan (10.407 min) Frag=170.0V 4720ABE.d
x10 4
305.0666
Mass=306
Monomer: gallocatechin
217.0034
141.0161
611.1392
881.1849
Counts vs. Mass-to-Charge (m/z)

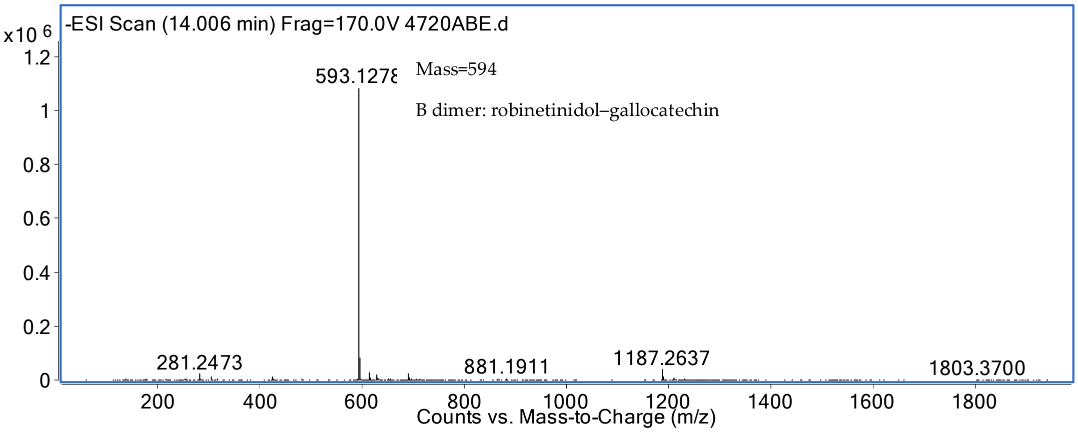
-ESI Scan (14.006 min) Frag=170.0V 4720ABE.d
x10 6
593.127
Mass=594
B dimer: robinetinidol–gallocatechin
281.2473
881.1911
1187.2637
1803.3700
Counts vs. Mass-to-Charge (m/z)

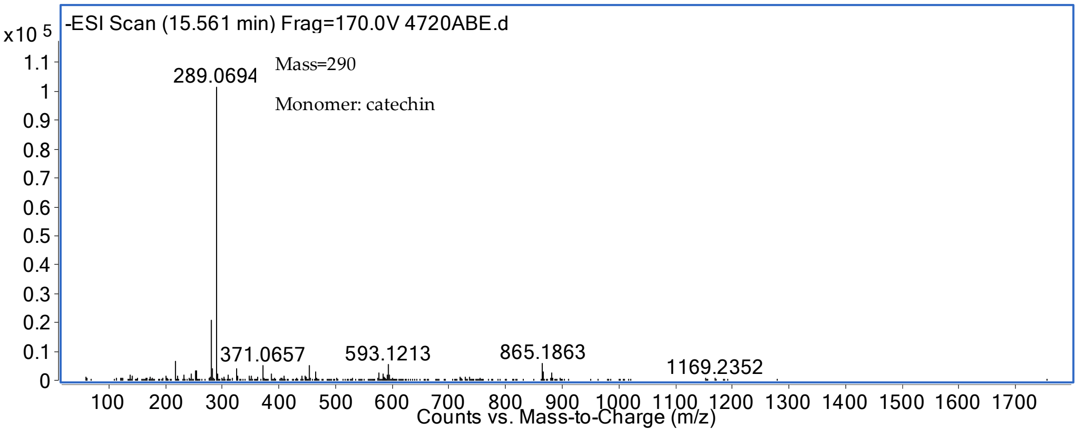
-ESI Scan (15.561 min) Frag=170.0V 4720ABE.d
x10 5
289.0694
Mass=290
Monomer: catechin
371.0657
593.1213
865.1863
1169.2352
Counts vs. Mass-to-Charge (m/z)

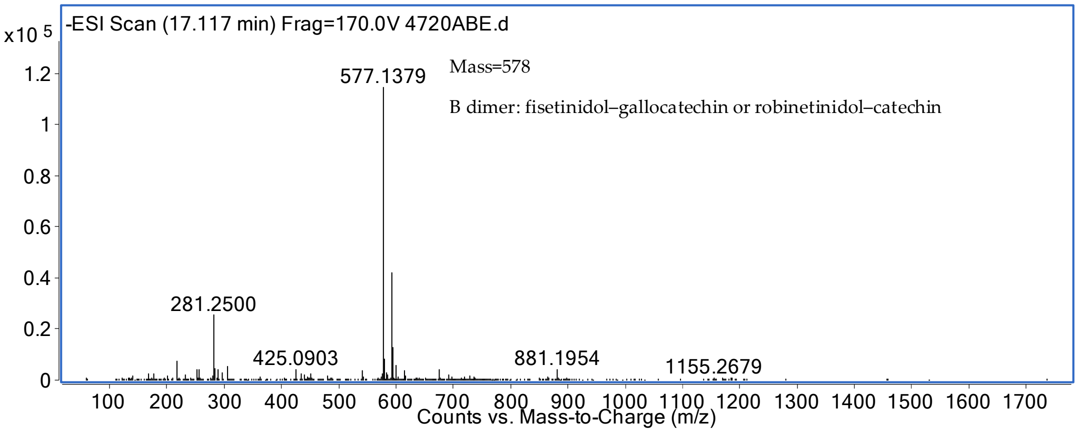
-ESI Scan (17.117 min) Frag=170.0V 4720ABE.d
x10 5
577.1379
Mass=578
B dimer: fisetinidol–gallocatechin or robinetinidol–catechin
281.2500
425.0903
881.1954
1155.2679
Counts vs. Mass-to-Charge (m/z)

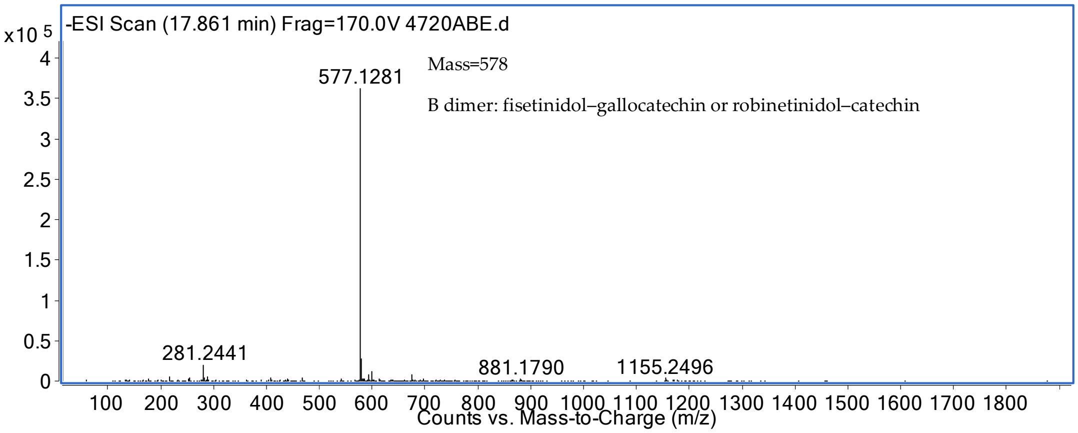
-ESI Scan (17.861 min) Frag=170.0V 4720ABE.d
x10 5
577.1281
Mass=578
B dimer: fisetinidol–gallocatechin or robinetinidol–catechin
281.2441
881.1790
1155.2496
Counts vs. Mass-to-Charge (m/z)

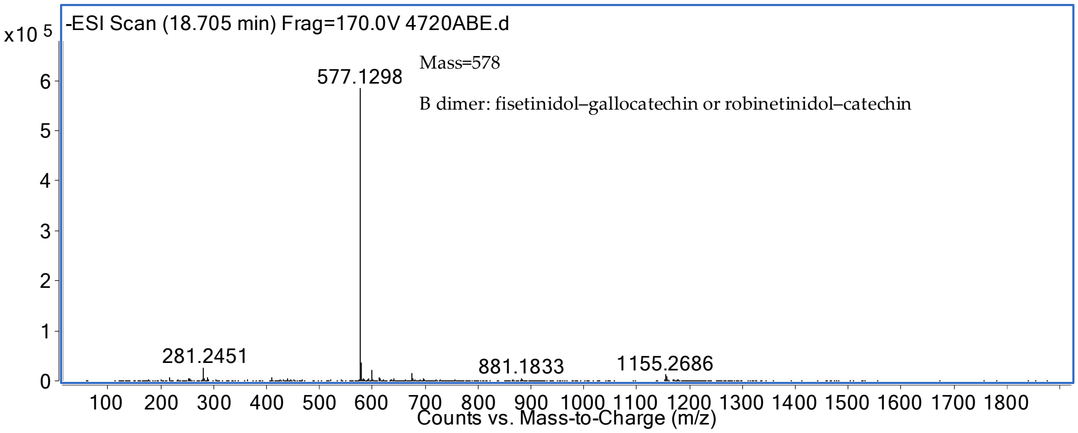
-ESI Scan (18.705 min) Frag=170.0V 4720ABE.d
x10 5
577.1298
Mass=578
B dimer: fisetinidol–gallocatechin or robinetinidol–catechin
281.2451
881.1833
1155.2686
Counts vs. Mass-to-Charge (m/z)

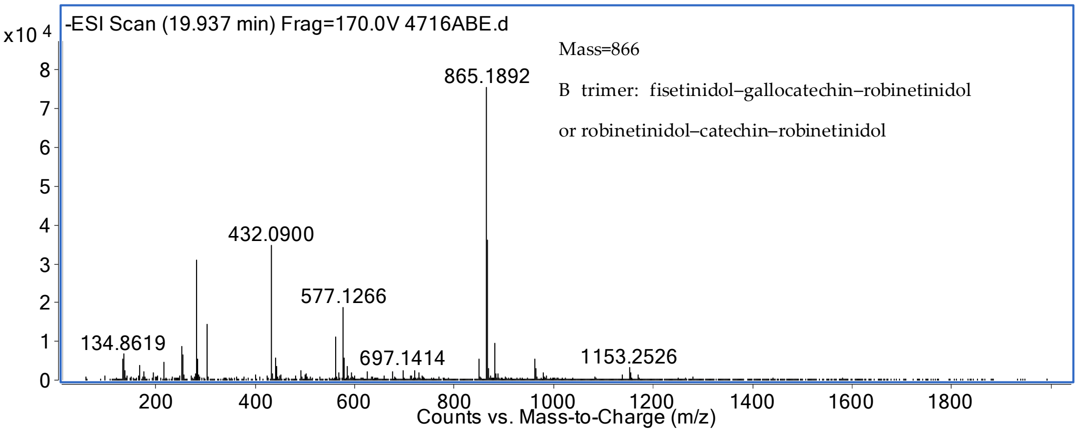
-ESI Scan (19.937 min) Frag=170.0V 4716ABE.d
x10 4
Mass=866
B trimer: fisetinidol–gallocatechin–robinetinidol
or robinetinidol–catechin–robinetinidol
865.1892
432.0900
577.1266
134.8619
697.1414
1153.2526
Counts vs. Mass-to-Charge (m/z)

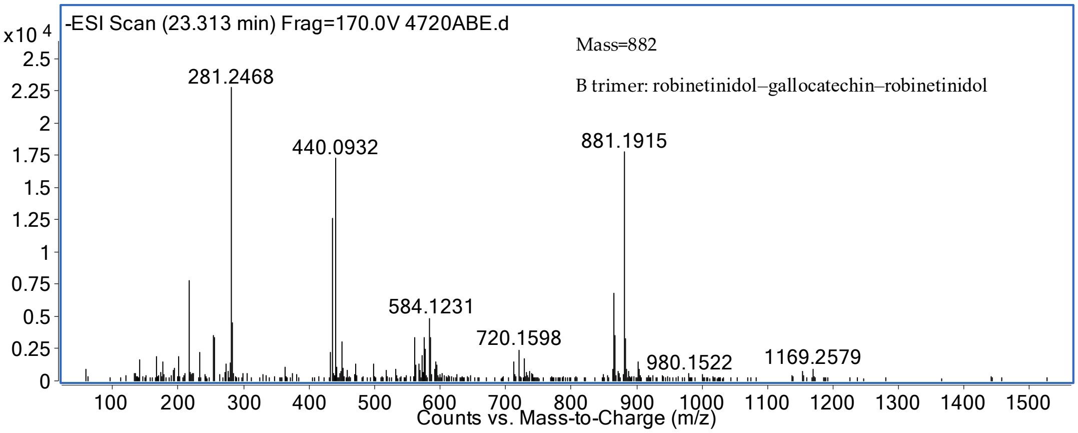
-ESI Scan (23.313 min) Frag=170.0V 4720ABE.d
x10 4
Mass=882
B trimer: robinetinidol–gallocatechin–robinetinidol
281.2468
440.0932
881.1915
584.1231
720.1598
980.1522
1169.2579
Counts vs. Mass-to-Charge (m/z)

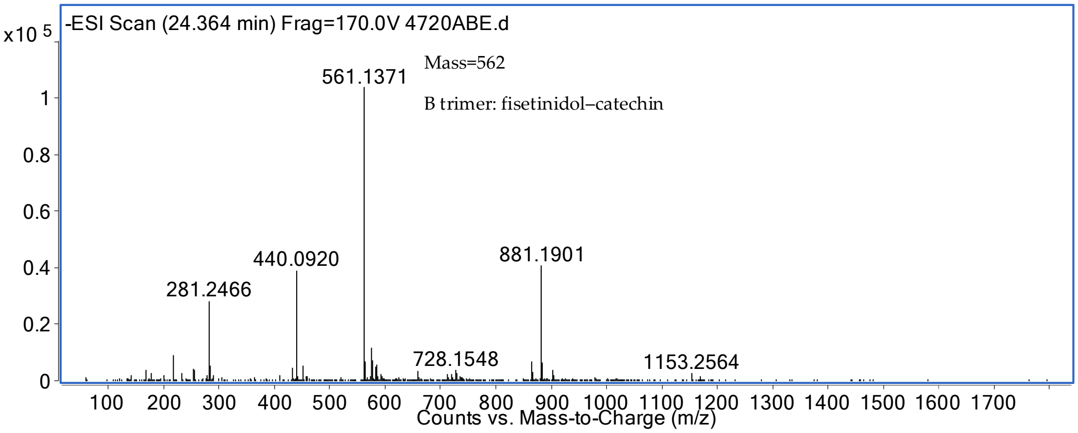
-ESI Scan (24.364 min) Frag=170.0V 4720ABE.d
x10 5
Mass=562
B trimer: fisetinidol–catechin
561.1371
440.0920
281.2466
881.1901
728.1548
1153.2564
Counts vs. Mass-to-Charge (m/z)

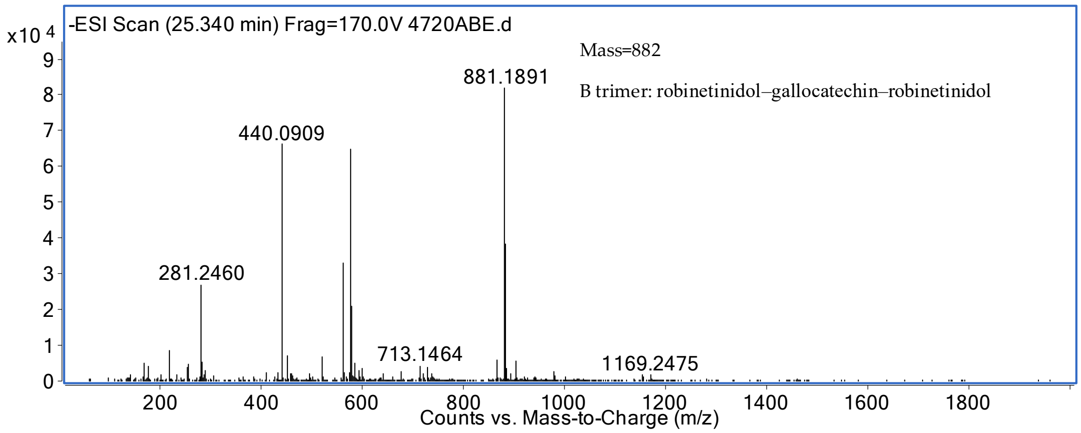
-ESI Scan (25.340 min) Frag=170.0V 4720ABE.d
x10 4
Mass=882
B trimer: robinetinidol–gallocatechin–robinetinidol
881.1891
440.0909
281.2460
713.1464
1169.2475
Counts vs. Mass-to-Charge (m/z)

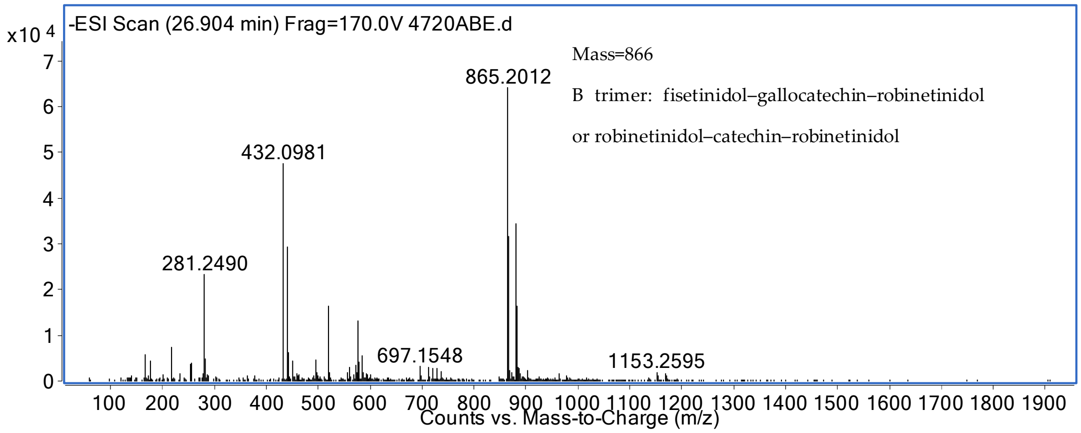
-ESI Scan (26.904 min) Frag=170.0V 4720ABE.d
x10 4
Mass=866
B trimer: fisetinidol–gallocatechin–robinetinidol
or robinetinidol–catechin–robinetinidol
865.2012
432.0981
281.2490
697.1548
1153.2595
Counts vs. Mass-to-Charge (m/z)

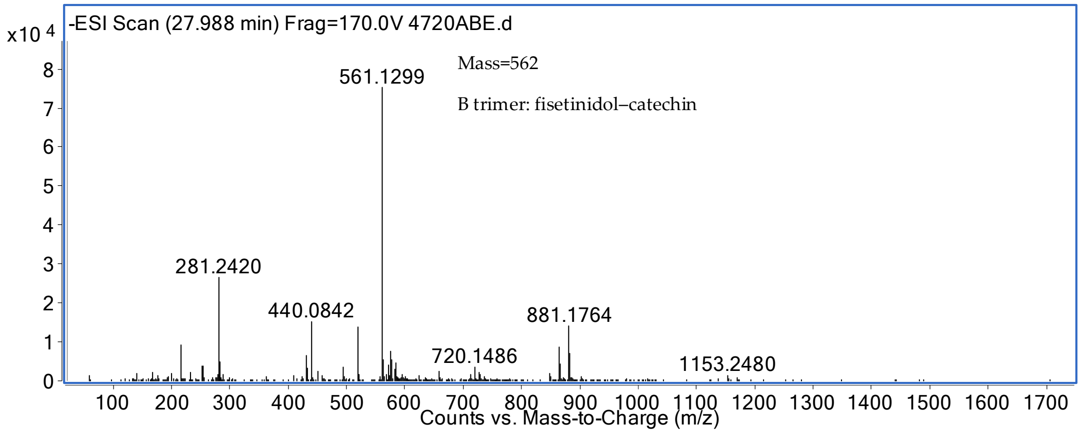
-ESI Scan (27.988 min) Frag=170.0V 4720ABE.d
x10 4
Mass=562
B trimer: fisetinidol–catechin
561.1299
281.2420
440.0842
881.1764
720.1486
1153.2480
Counts vs. Mass-to-Charge (m/z)

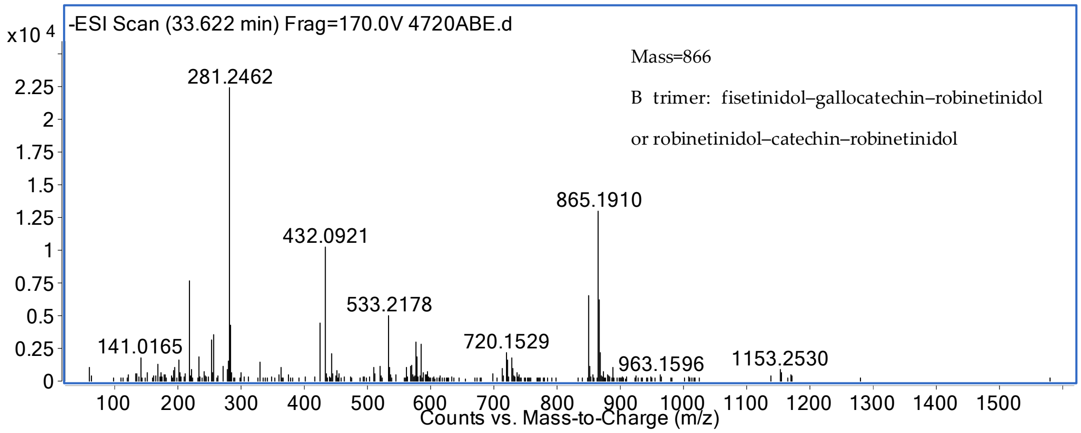
-ESI Scan (33.622 min) Frag=170.0V 4720ABE.d
x10 4
Mass=866
B trimer: fisetinidol–gallocatechin–robinetinidol
or robinetinidol–catechin–robinetinidol
281.2462
865.1910
432.0921
533.2178
720.1529
141.0165
963.1596
1153.2530
Counts vs. Mass-to-Charge (m/z)

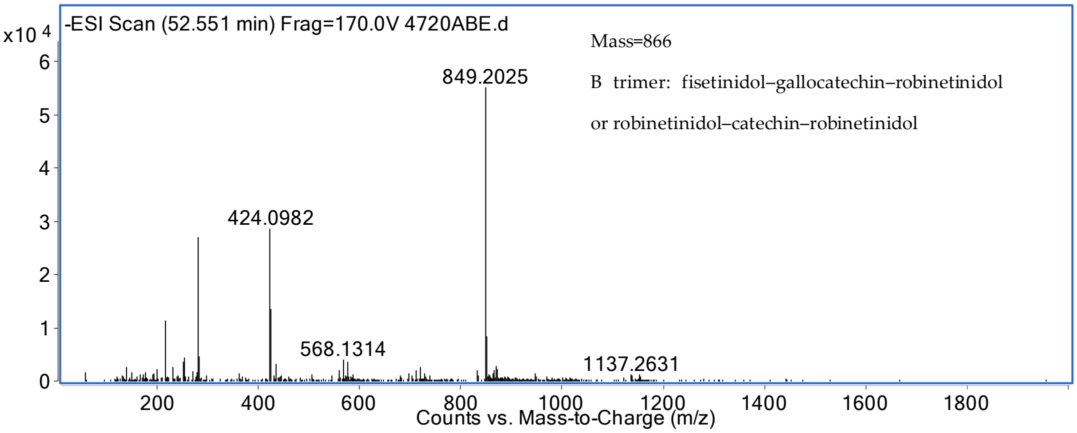

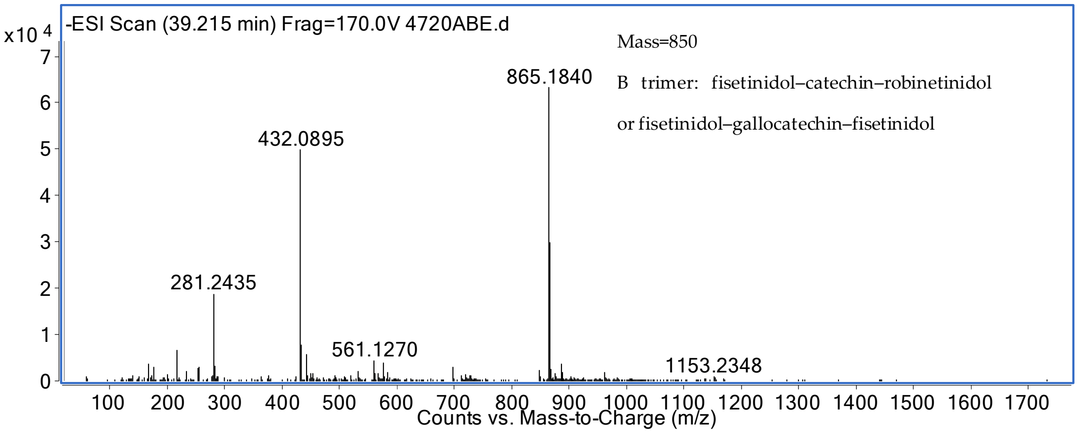

Figures S1. Mass spectram and proposed copounds of proanthocyanidins from *Acacia mearnsii* bark under different retention time.

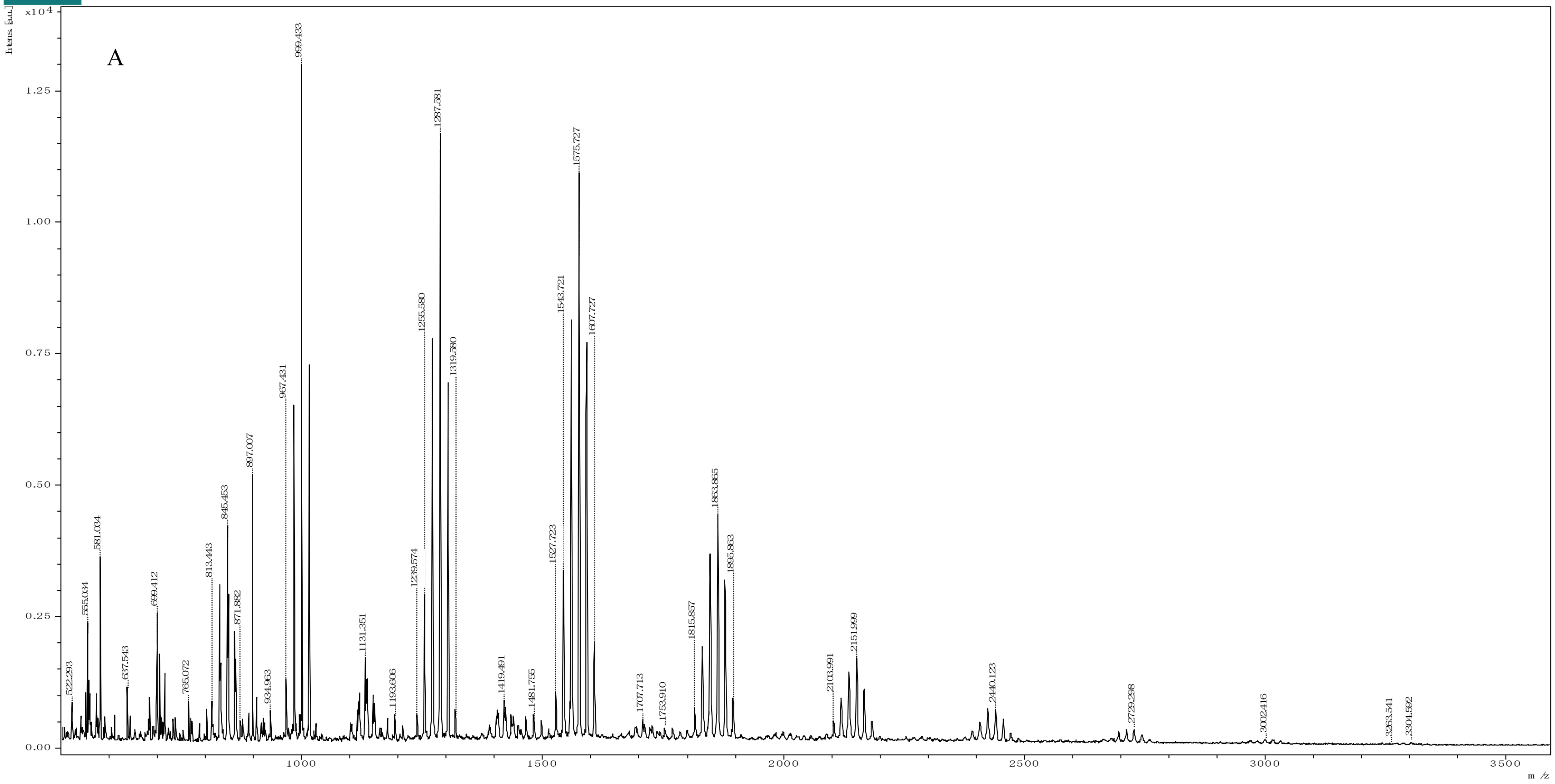
A
x10^4
Intens. [a.u.]
0.00
0.25
0.50
0.75
1.00
1.25
1000
1500
2000
2500
3000
3500
m/z
522.293
555.034
581.034
637.543
699.412
765.072
813.443
845.453
871.882
897.007
934.963
967.431
999.433
1131.351
1193.606
1239.574
1255.580
1287.581
1319.580
1419.491
1481.755
1527.723
1543.721
1575.727
1607.727
1707.713
1753.910
1815.857
1863.865
1895.863
2103.991
2151.999
2440.123
2729.298
3002.416
3253.541
3301.592

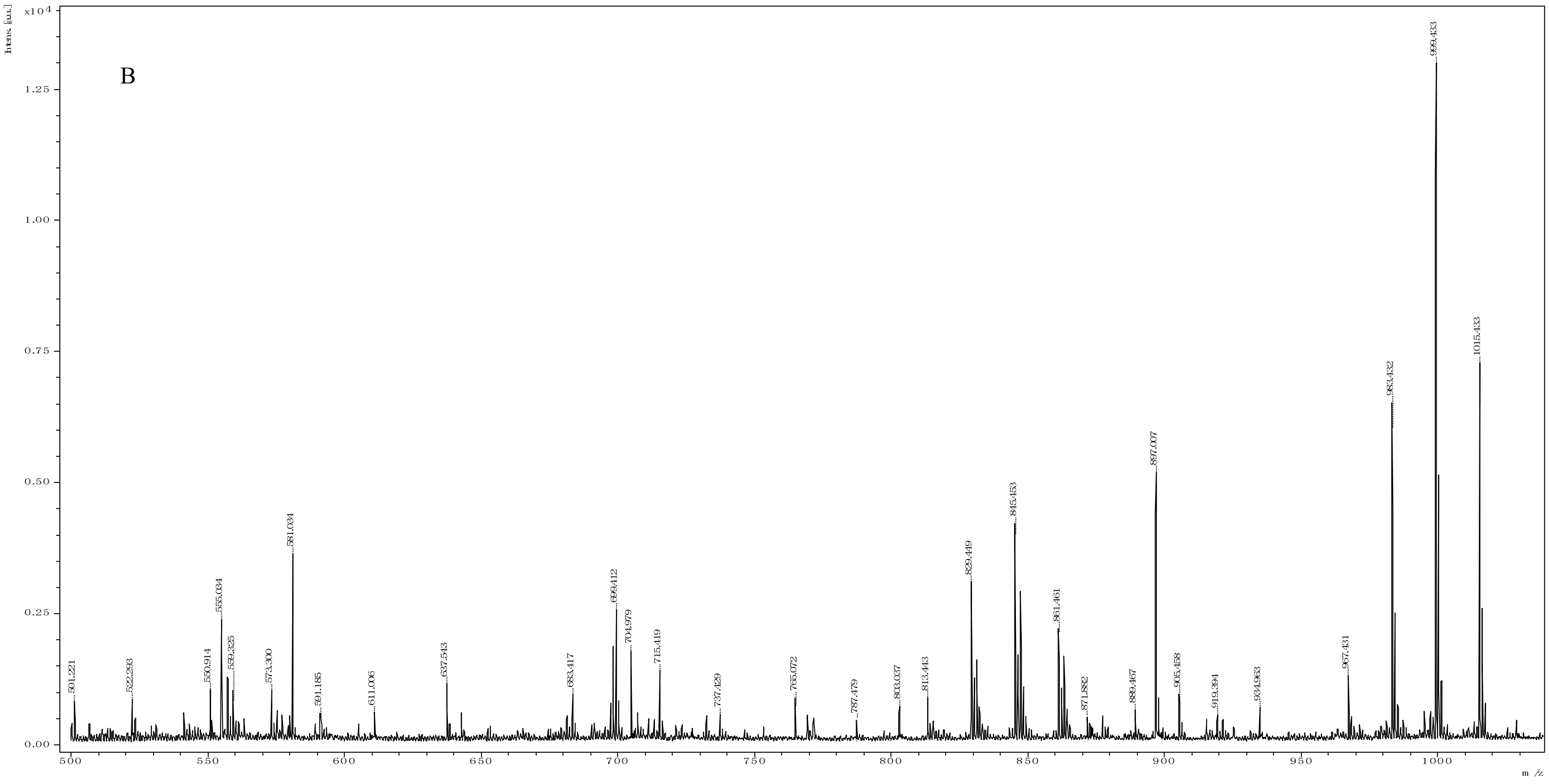
B
Intens. [a.u.]
x10⁴
m/z
501.221
522.293
550.914
555.034
559.325
573.300
581.034
591.185
611.006
637.543
683.417
699.412
704.979
715.419
737.429
765.072
787.479
803.037
813.443
829.449
845.453
861.461
871.882
889.467
897.007
905.458
919.394
934.963
967.431
983.432
999.433
1015.433

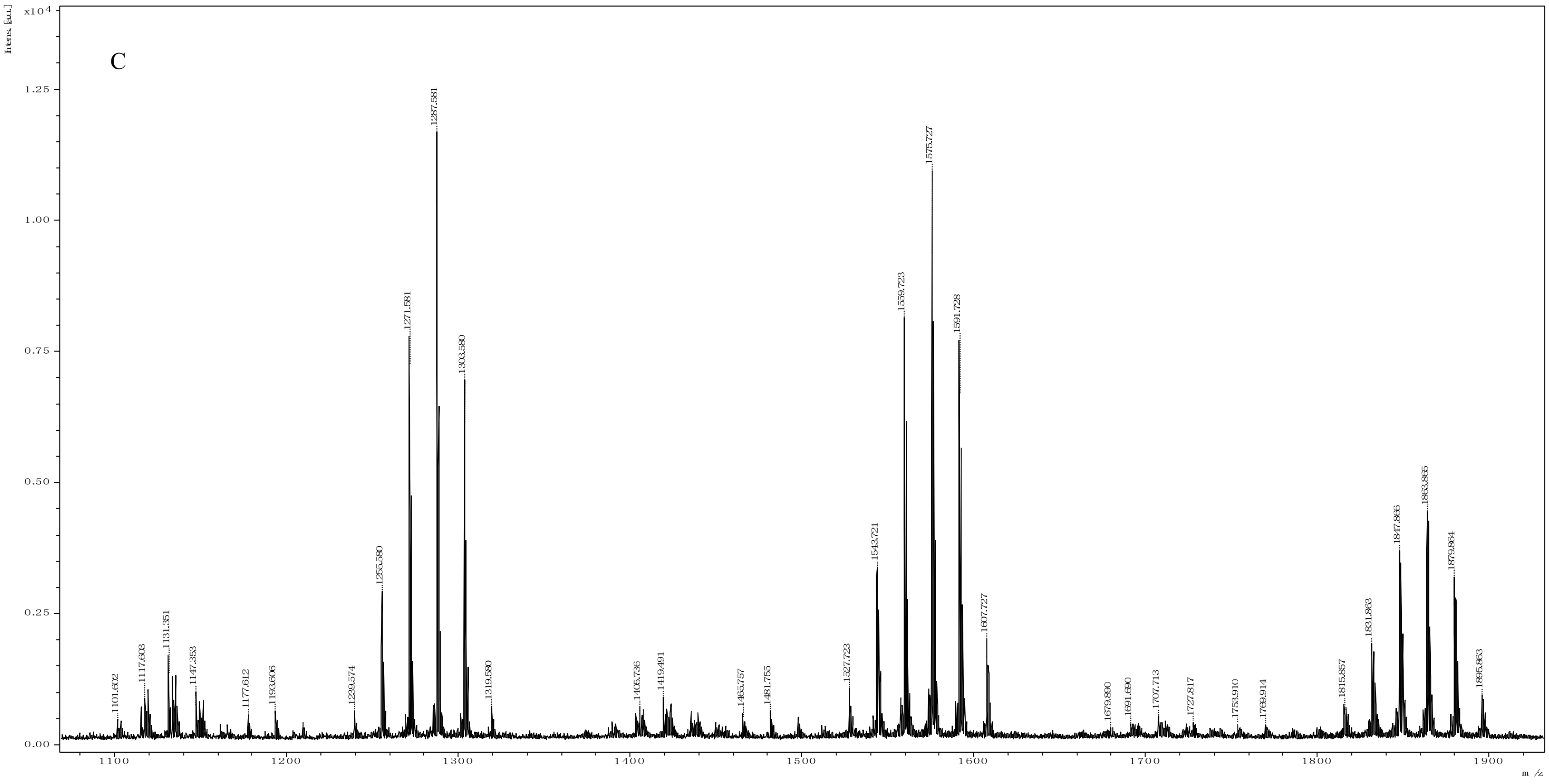
C
Intens. [a.u.]
x10^4
1101.602
1117.603
1131.351
1147.353
1177.612
1193.606
1239.574
1255.580
1271.581
1287.581
1303.580
1319.580
1405.736
1419.491
1465.757
1481.755
1527.723
1543.721
1559.723
1575.727
1591.728
1607.727
1679.890
1691.690
1707.713
1727.817
1753.910
1769.914
1815.857
1831.863
1847.866
1863.865
1879.864
1895.863
m/z

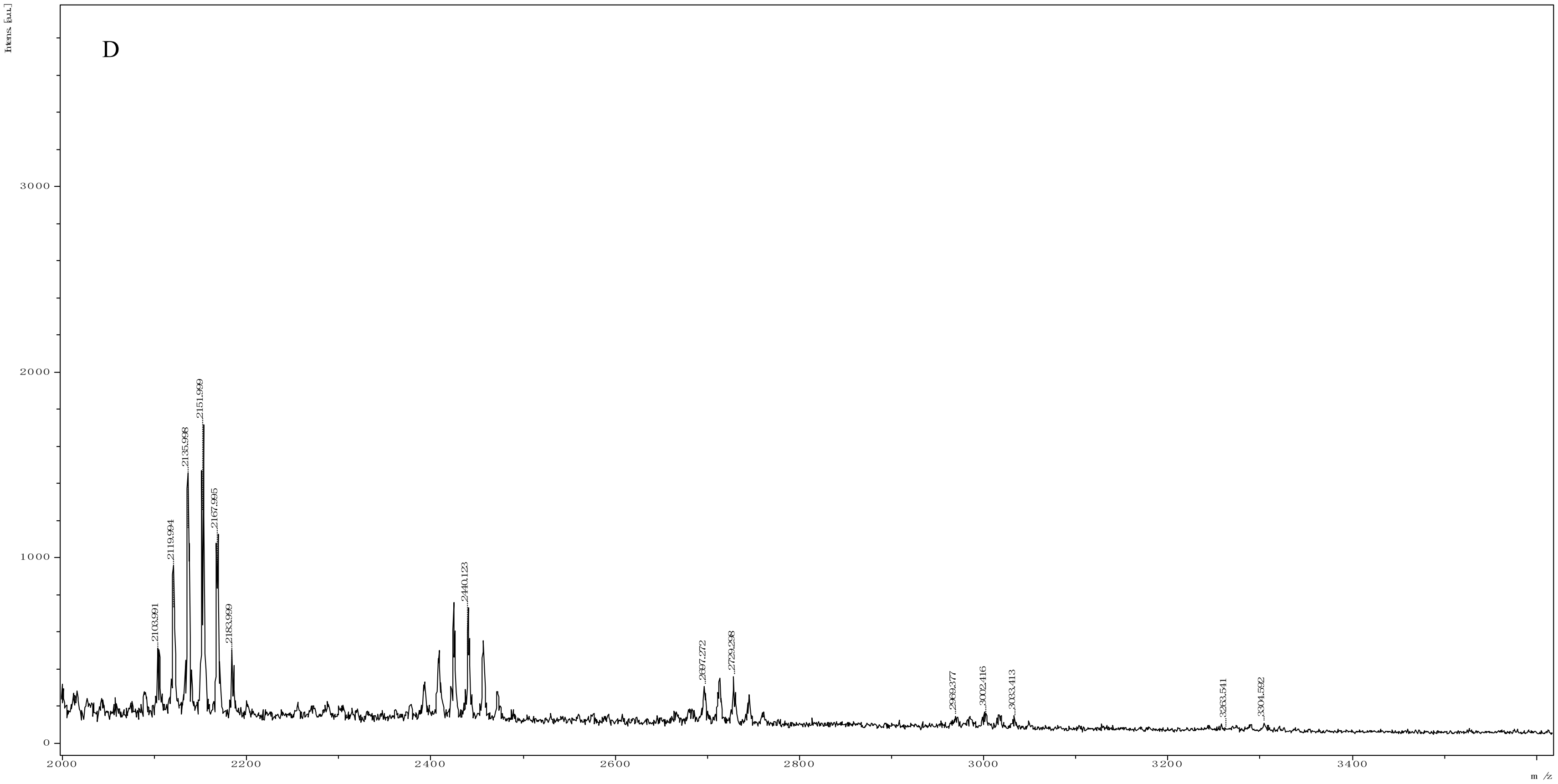

Figures S2. MALDI-TOF positive reflectron mode mass spectra of ABE: full spectrum (A), details of the 500-1000 m/z (B), 1100-1900 m/z (C), and 2000-3600 m/z (D).
